# Characterisation of the Interaction of the C-Terminus of the Dopamine D2 Receptor with Neuronal Calcium Sensor-1

**DOI:** 10.1371/journal.pone.0027779

**Published:** 2011-11-16

**Authors:** Lu-Yun Lian, Sravan R. Pandalaneni, Pryank Patel, Hannah V. McCue, Lee P. Haynes, Robert D. Burgoyne

**Affiliations:** 1 NMR Centre for Structural Biology, Institute of Integrative Biology, University of Liverpool, Liverpool, United Kingdom; 2 The Physiological Laboratory, Department of Cellular and Molecular Physiology, Institute of Translational Medicine, University of Liverpool, Liverpool, United Kingdom; Griffith University, Australia

## Abstract

NCS-1 is a member of the neuronal calcium sensor (NCS) family of EF-hand Ca^2+^ binding proteins which has been implicated in several physiological functions including regulation of neurotransmitter release, membrane traffic, voltage gated Ca^2+^ channels, neuronal development, synaptic plasticity, and learning. NCS-1 binds to the dopamine D2 receptor, potentially affecting its internalisation and controlling dopamine D2 receptor surface expression. The D2 receptor binds NCS-1via a short 16-residue cytoplasmic C-terminal tail. We have used NMR and fluorescence spectroscopy to characterise the interactions between the NCS-1/Ca^2+^ and D2 peptide. The data show that NCS-1 binds D2 peptide with a K_d_ of ∼14.3 µM and stoichiometry of peptide binding to NCS-1 of 2∶1. NMR chemical shift mapping confirms that D2 peptide binds to the large, solvent-exposed hydrophobic groove, on one face of the NCS-1 molecule, with residues affected by the presence of the peptide spanning both the N and C-terminal portions of the protein. The NMR and mutagenesis data further show that movement of the C-terminal helix 11 of NCS-1 to fully expose the hydrophobic groove is important for D2 peptide binding. Molecular docking using restraints derived from the NMR chemical shift data, together with the experimentally-derived stoichiometry, produced a model of the complex between NCS-1 and the dopamine receptor, in which two molecules of the receptor are able to simultaneously bind to the NCS-1 monomer.

## Introduction

The neuronal calcium sensor (NCS) proteins are EF-hand containing Ca^2+^- binding proteins that detect Ca^2+^ signals to regulate a wide range of cellular functions in neurons, photoreceptor cells and other cell types [1]. The NCS family consists of proteins encoded by 14 genes in mammalian species and there also additional splice variants. The family can be divided into five groups consisting of NCS-1, the neurocalcins/VILIPs (visinin-like proteins), recoverin, GCAPs (guanylate cyclase activating proteins) and the KChIPs (K^+^ channel interacting proteins) [1]. Despite there being high levels of sequence similarity between the NCS proteins, genetic studies have indicated that they have specific non-overlapping functions that cannot be compensated for by other family members [2], [3]. The specificity of their physiological roles depends in part on specific interaction with and regulation of distinct target proteins.

One member of the NCS family, NCS-1, has orthologues from *Saccharomyces cerevisiae* (Frq1) [4] to man and has been implicated in several physiological functions including regulation of neurotransmitter release [5], [6], membrane traffic [7], voltage gated Ca^2+^ channels [8], [9], [10], neuronal development [11], [12], synaptic plasticity [13], [14] and learning [15], [16]. NCS-1 is N-terminally myristoylated which allows its association with distinct membrane compartments including the plasma membrane and the trans-Golgi network [17] and it cycles between membrane-bound and cytosolic pools [18]. It has been shown to interact with a wide range of target proteins [19], [20] including phosphatidylinositol-4-kinase (PI4K) IIIβ [7], [21] and its orthologue Pik1 in yeast [4], ARF1 [7], [22], interleukin receptor accessory protein like-1 (IL1RAPL1)[23], TRPC5 channels [11], InsP(3) receptors [24] and the dopamine D2 and D3 receptors [25].

The interaction of NCS-1 with the dopamine D2 receptor is potentially of considerable interest considering the key physiological role of dopamine signalling within the CNS and its implication in addictive behaviour [26]. In addition the D2 receptor subtype is the target for most antipsychotic drugs [27] and such drugs affect the expression levels of NCS-1 [28]. The D2 receptor possesses a short 16 residue intracellular C-terminal domain and in a yeast 2-hybrid screen this was found to bind to NCS-1. The functional effect of the NCS-1-D2 interaction was shown to be inhibition of the phosphorylation and subsequent internalisation of D2 receptors following ligand binding in a heterologous cell line [25]. More recently a physiological role for NCS-1 in controlling dopamine D2 receptor surface expression and thereby in modifying synaptic plasticity and exploratory behaviour has been established in mice [16].

**Figure 1:**
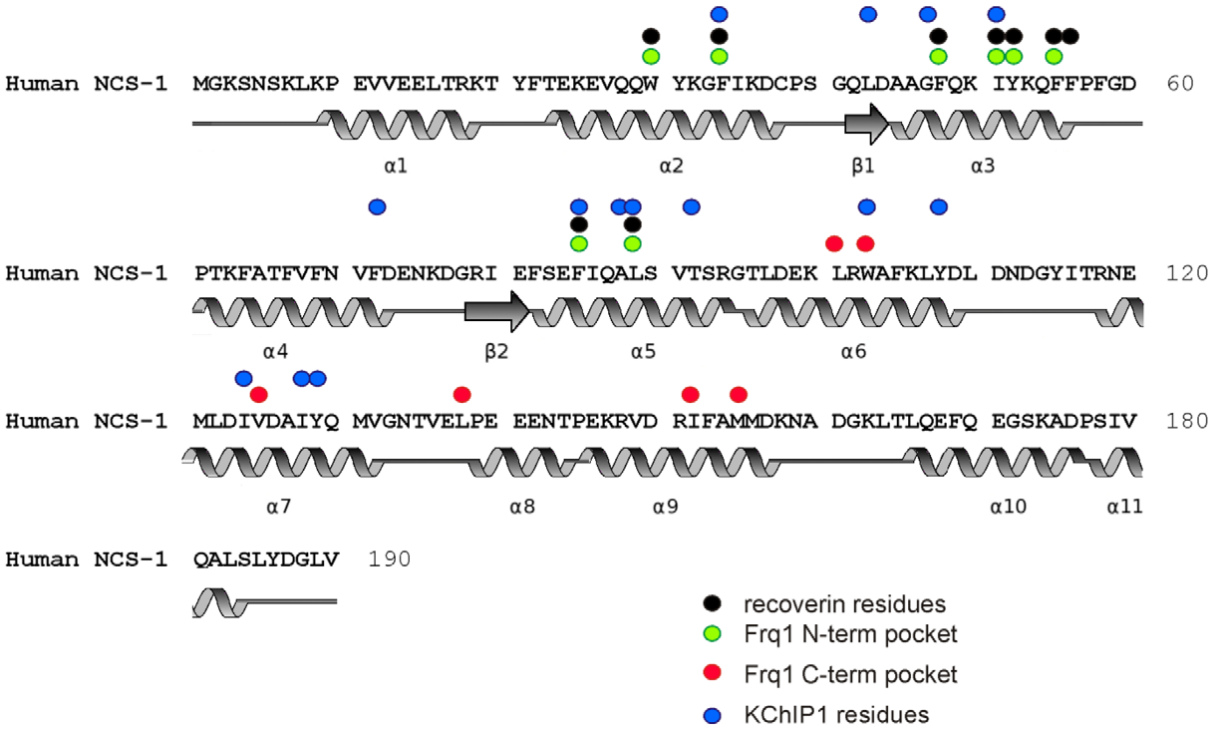
Conserved hydrophobic residues involved in protein target interactions in NCS proteins. The secondary structure elements indicated are obtained from the crystal structure of human NCS-1 (1G8I). The region between 134–138 is either unstructured or form a short helix; the latter is indicated in this figure. The residues identified to make contact with target peptides in the recoverin/rhodopsin kinase [33], Frq1/Pik1 [31] and KChIP1/Kv4 [35] complexes are indicated above the corresponding amino acids in NCS-1 which are all conserved hydrophobic residues. For the Frq1/Pik1 complex residues are indicated that interact with the two alpha helices of Pik1 n the N- and C-terminal parts of the hydrophobic groove.

The molecular basis for the interaction of NCS-1 with the C-terminus of the dopamine D2 receptor has been speculated upon [29] but has not been established through structural investigations. Structures of several NCS proteins have been determined by use of X-ray crystallography or NMR spectroscopy including for NCS-1 [30]. In addition, the structures of three complexes of NCS proteins with fragments of target proteins have been solved. These are for *S. cerevisiae* Frq1 with a fragment of Pik1 [31] (and a very similar complex for the equivalent proteins from Schizo*saccharomyces pombe* [32]), recoverin with an N-terminal fragment of rhodopsin kinase [33] and KChIP1 with an N-terminal domain of the Kv4.3 potassium channel [34], [35]. In each case the target peptide was bound within a solvent-exposed hydrophobic cleft in the Ca^2+^-loaded form of the NCS protein. The cleft was formed from hydrophobic residues conserved in all of the NCS family members (Figure 1) suggesting that a similar mode of target binding could be used by all of the NCS proteins although variation in the size of the exposed cleft and surface area available for the interaction with different target proteins could contribute to target specificity.

A large hydrophobic cleft was exposed in Ca^2+^-bound NCS-1 in the crystal structure suggesting that this could be the site of protein ligand interaction [30]. Surprisingly, however, data from the use of yeast 2-hybrid screening suggested that the D2 receptor C-terminus could interact with the extreme N-terminus (residues 1–71) of NCS-1 when expressed alone. This finding could indicate a different mode of target interaction to that seen for other NCS protein-target interactions. In order to determine if this is indeed the case and whether this interaction could provide a specific target for drug action, we have set out to further characterise the interaction of the D2 receptor C-terminus with NCS-1 by use of fluorescence spectrophotometry and NMR spectroscopy. The data suggest that D2 peptide interacts with NCS-1 in a 2∶1 peptide: protein stoichiometry, and that the peptide binds to the large hydrophobic groove on one face of the NCS-1 molecule. The data further show that movement of the C-terminal helix of NCS-1 to fully expose the hydrophobic groove may be important for D2 peptide binding.

## Materials and Methods

### Peptide synthesis

The peptide (hereafter referred to as D2 peptide) used here corresponds to residues 428–443 of the human D2 receptor. The synthetic D2 peptide (TFNIEFRKAFLKILHC) and its modified form D2SR (TFNIEFRKAFLKILHSR), were purchased from Peptide Protein Research Ltd., Fareham, PO15 6DP, UK. The shorter form, corresponding to residues 428–435 (TFNIEFRK) were, purchased from GenicBio, China. The peptides was delivered >95% pure. Both D2 and D2SR peptides were rather insoluble in aqueous buffer. DMSO was found to be the most suitable organic solvent for the initial solubilisation of the peptide. Therefore, in all the experiments, peptide samples were first dissolved in a minimal amount of DMSO and then diluted with the relevant buffer. If necessary, pH values of the samples were re-adjusted to the desired value.

### Plasmid production

An expression construct of *Rattus norvegicus* NCS-1 [6] was sub-cloned from a pGEX-6p plasmid expressing NCS-1 [36] into pET-M11 vector. The primers were designed based on the cDNA sequence obtained from GenBank accession no. L27421 with Nco-1 and Kpn-1 as restriction digest sites. The sense and antisense strands were 5′GGCGCCATGGGGAAATCCAACAGCAAGTTG 3′ and 5′CGGATCCGGTACCTTA CTA TAC CAG CCC GTC GTA 3 ′ respectively. Polymerase chain reaction was carried out using KOD Hot Start DNA Polymerase from Novagen. Confirmation of the insert was obtained through DNA sequencing. The R102Q mutation [18] was generated by site-directed mutagenesis.

### Protein expression and purification

NCS-1 plasmid was transformed into E.*coli* strain BL21 (DE3) (Novagen). Protein expression in Luria Broth (LB) and 2M9 minimal media performed by growing the cells to mid log (0.6–0.7 OD A^o^600) at 30^°^C; after induction with 1 mM IPTG the culture was left to grow overnight at 18^°^C. ^15^N NCS-1 and doubly labelled ^13^C^15^N proteins were expressed using 2M9 media containing ^15^NH_4_Cl only or ^13^C glucose and ^15^NH_4_Cl as the carbon and nitrogen source. 1 ml of the starter culture was grown in LB overnight, centrifuged, and the pellet added to a 50 ml labelled media and left to grow overnight in 50 ml labelled culture. The overnight culture was used to inoculate the labelled growth medium so that the starting optical density was less than 0.1 A-600 and protein expressed as described above. Cells were harvested and resuspended into the lysis buffer (50 mM TrisHCl, 200 mM NaCl 5 mMCaCl_2_ pH 7.5 plus a tablet per litre of culture of Complete EDTA Free Protease inhibitor (Roche Applied Science).

The cells were lysed using a French Press. Deoxyribonuclease I (250 µg) from bovine pancreas (Sigma) was added to the cell lysate and the lysate was centrifuged at 20 K RPM (47,807 g) for 30 mins. The supernatant was collected and filtered through 0.22 µ acrodisc and loaded on to the Hiprep 16/10 Phenyl FF High Sub (GE Healthcare) column that had been pre-equilibrated with Buffer-A (50 mM TrisHCl, 200 mM NaCl and 5 mM CaCl_2_ pH 7.5). Unbound proteins were removed through extensive wash with Buffer A. A second wash with Buffer B (50 mM TrisHCl, 0.5 mM CaCl_2_ pH 7.5) removed further impurities. NCS-1 was eluted from the column with MilliQ water. Eluted protein was buffer exchanged into Tobacco Etch Virus (TEV) protease cleavage buffer (50 mM Tris 500 mM NaCl pH 7.4) and the N-terminal his tag cleaved overnight at 4^°^C using a 1∶20 molar ratio of TEV protease:NCS-1. The cleaved protein was further purified using Superdex 75 Hiload 26/60 (Amersham Biosciences) size exclusion column (50 mM TrisHCl, 150 mM NaCl pH 7.5). The yield from one litre of culture was between 50–100 mgs. The purity of the NCS-1 was confirmed by SDS-PAGE. Confirmation of the identity of the purified NCS-1 was obtained through Maldi-tof mass spectrometry. Analysis of the protein was carried out using Size Exclusion Chromatography Multi-angle Laser Light Scattering (SEC-MALS) which indicated that NCS-1 was monomeric.

### Spectrofluorometry

To monitor intrinsic tryptophan fluorescence of NCS-1 proteins [36], [37], purified recombinant NCS-1 at a concentration of 1 µM in a calcium free buffer (20 mM HEPES, 139 mM NaCl, 2 mM ATP, 5 mM EGTA, 5 mM nitrolotriacetic acid, pH 7.4) were excited at room temperature with 280 nm light, and their emission spectra from 290–410 nm were measured, using a Jasco FP-6300 spectrofluorometer (Tokyo, Japan). The concentration of free calcium was increased by addition of CaCl_2_ to give a calculated free Ca^2+^ concentration of 1 µM and an additional emission spectrum were measured. The D2 peptide was then added to give incremental increase in peptide concentration with emission spectra measured after each addition. The data for the measured fluorescence change at each peptide concentration were fitted to a logistic fit using non-linear curve fitting in OriginPro (Microcal).

### NMR Spectroscopy

NCS-1 was prepared in Tris buffer pH 6.8 in the presence of 5 mM MgCl_2_ and 5 mM CaCl_2_. Unless otherwise state, NMR spectra were recorded at 27 °C on Bruker DRX 800 and 600 MHz spectrometers equipped with CryoProbes. Data were processed using the Bruker Software TopSpin and analysed using CCPN software [38]. Sequence-specific backbone and side-chain resonance assignment of NCS-1 was transferred from the previously reported assignments [39] on unmyristoylated Ca^2+^ loaded NCS-1 (Biological Magnetic Resonance Data Bank accession number 4378); however, due to differences in sample conditions and temperature, many resonances were re-assigned during the course of these studies using HNCA, HN(CO)CA, HNCO, HN(CA)CO, CBCA(CO)NH, CBCANH, HBHA(CO)NH and HCCH-TOCSY experiments. In addition, the resonance assignment for the aromatic side-chain had been incomplete. The aromatic side-chains were partially assigned using 2D [^1^H, ^13^C] -TROSY, and homonuclear TOCSY and NOESY spectra recorded in D_2_O.

Two peptides D2 and D2SR were used for the NMR studies. Secondary structure prediction showed that the addition of the two extra amino acids did not significantly affect the helical content of the D2 peptide but in theory could increase solubility, although in practice the solubility improvement was only marginal. Both peptides had similar effects on the NCS-1 NMR spectrum. Since D2SR did not have the complications of cysteine oxidation over prolonged NMR experiments, the data from this peptide was used for the final analyses. The peptides were first dissolved in minimal amounts of DMSO (approximately 1–2 µl) before adding the protein solution to achieve the appropriate peptide to protein ratio.

The minimum chemical-shift differences between free and D2-bound NCS-1 were expressed as: Δδ = √ {(ΔH/0.03)^2^ + (ΔN/0.03)^2^}. Values of Δδ greater than 0.07 were considered significant.

### NCS1-D2 and NCS-1-D3 complex modelling

Due to exchange broadening, attempts to obtain Intermolecular NOEs between ^15^N ^13^C labelled NCS-1 and unlabelled peptide using filtered NOESY experiments [40] were unsuccessful. Peptide-protein docking utilised the restraints-driven docking Webserver programme HADDOCK 2.1 (*H*igh *A*mbiguity *D*riven biomolecular *DOCK*ing) [41]. For these docking experiments, the known structures of the human NCS-1 (1G8I) with the coordinates for the extreme C-terminal removed and coordinates for the C-terminus peptide TFNIEFRKAFLKILSC of the D3 receptor (residues residues 385–400 from the crystal structure of the dopamine D3 receptor (PDB Accession 3PBL) were used. The C-terminus of D3 differs from the D2 receptor by only the penultimate amino acid (His in D2 rather that Ser in D3). Active residues used as the Ambiguous Interaction Restraints (AIR) included NCS-1 residues whose ^15^N-^1^H and ^13^C-^1^H shifts were significantly perturbed in the presence of D2SR. The hydrophobic residues of D2SR which could be involved with NCS-1 binding were identified through sequence and structural alignment with Pik 1 [31]. The HADDOCK derived structures satisfied the restraints provided and the D2/D3 peptide docked into the two hydrophobic grooves previously identified in [31]. To obtained a model of the complex with the whole D3 receptor, a similar approach was employed but using the coordinates from the receptor rather than only the C-terminal polypeptide. AIR restraints for NCS-1, based on the chemical shift mapping data, were similar to those used to obtain the protein-peptide complex. Flexibility was defined for the receptor region between V381–F386 to allow for movement in the C-terminal region (equivalent to the D2 peptide). The docking results were automatically analysed by the HADDOCKprogramme to provide the cluster of structures which were deemed the most likely structures. 200 water-refined structures were calculated and clustered together according to the ligand interface RMSD. The RMSD cut-off for clustering was 7.5 Angstrom. Individual clusters were analysed and the RMSD to lowest energy structure, the buried surface area per cluster and various energy terms (van der Waals, electrostatics) was calculated. Where more than one cluster was generated, the cluster of structures with a combination of the lowest energy and highest buried surface area was used as the top ranking structures.

## Results

### Affinity and stoichiometry of D2 peptide binding to NCS-1

**Figure 2:**
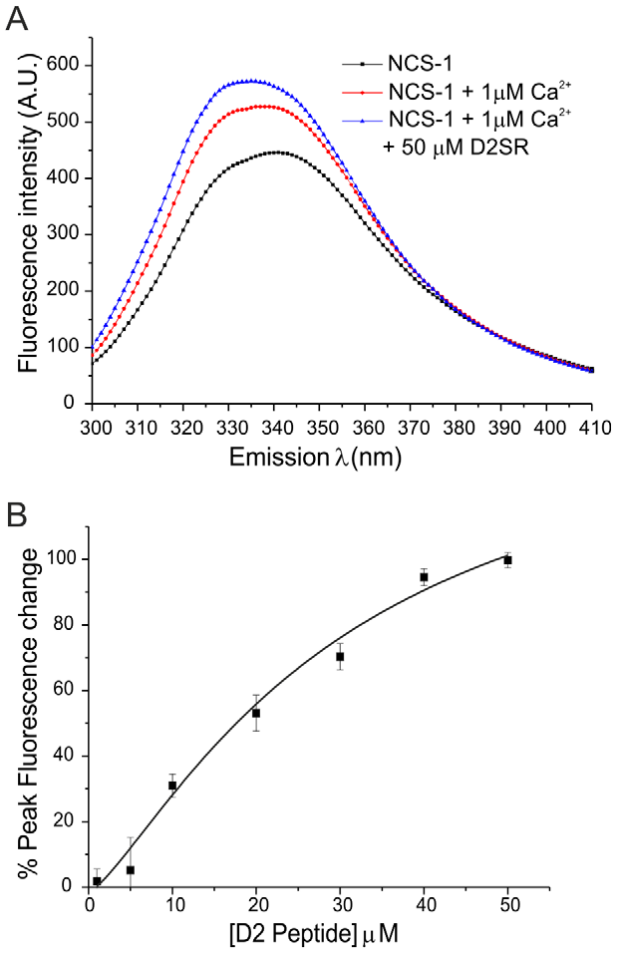
Binding of the dopamine D2 receptor C-terminus to NCS-1 monitored using tryptophan fluorescence. (A) The spectra represent intrinsic tryptophan fluorescence of NCS-1 before and after addition 1 µM Ca^2+^ and after subsequent addition of the D2SR peptide. (B) Extent of the changes in tryptophan fluorescence following sequential additions of D2SR peptide to the final concentrations indicated. The data are shown as mean±SEM (n = 5) and were fitted using a logistic fit by non-linear curve fitting.

To determine if the binding of the dopamine D2 receptor C-terminus to NCS-1 could be examined in solution we initially used a 16 residue synthetic peptide corresponding to human D2 (428–443) or a modified peptide D2SR designed for increased solubility. To assay binding we measured the intrinsic tryptophan fluorescence of NCS-1. The protein has two tryptophan residues and changes in their fluorescence occurs due to conformational changes following Ca^2+^-binding [37], [42]. D2 peptide addition resulted in an increase in fluorescence in the presence or absence of Ca^2+^ but there was apparently lower affinity for peptide in Ca^2+^-free conditions. Further analysis was carried out, therefore, in the presence of 1 µM free Ca^2+^. Addition of either the D2 or the D2SR peptide resulted in an additional increase in fluorescence of a similar magnitude above that following the addition of 1 µM free Ca^2+^ and allowed titration of the binding of the peptide over a range of concentrations (Figure 2A). The data for the change in fluorescence versus concentration was fitted with a logistic fit. In initial experiments with the D2 peptide the dose-response indicated half-maximal binding at 14.3 µM with a Hill coefficient of 2.1. From more detailed analyses with the D2SR peptide the dose-response was fitted to give half-maximum binding at 30.16+5.97 µM (Figure 2B). The binding curves from the experiments consistently indicated a Hill coefficient close to 1.5. Hill coefficients greater than 1 would be consistent with a 2∶1 stoichiometry of peptide binding to NCS-1 since NCS-1 itself was monomeric, as assessed by size exclusion chromatography and multi-angle light scattering (unpublished data).

A yeast 2-hybrid analysis [25] suggested that the shortest peptide that could bind NCS-1 corresponded to residues 428–435 (TFNIEFRK) of the receptor. This seems surprising as these are the cytoplasmic residues in closest proximity to the transmembrane domain of the receptor and that in the structure of the dopamine D3 receptor (PDB accession 3PBL), the first two amino acids TF form part of penultimate helix VII of the receptor, rather than the C-terminus helix VIII. Nevertheless we tested the ability of this shorter peptide to bind to NCS-1 but could find no evidence for an interaction using both fluorescence and NMR titrations using up to a 10-fold excess of peptide. Preliminary data also shows that the peptide NIEFRKAFLKILHS is able to bind NCS-1 with similar affinity to TFNIEFRKAFLKILHS.

### Identification of the D2 peptide binding site by NMR spectroscopy

**Figure 3:**
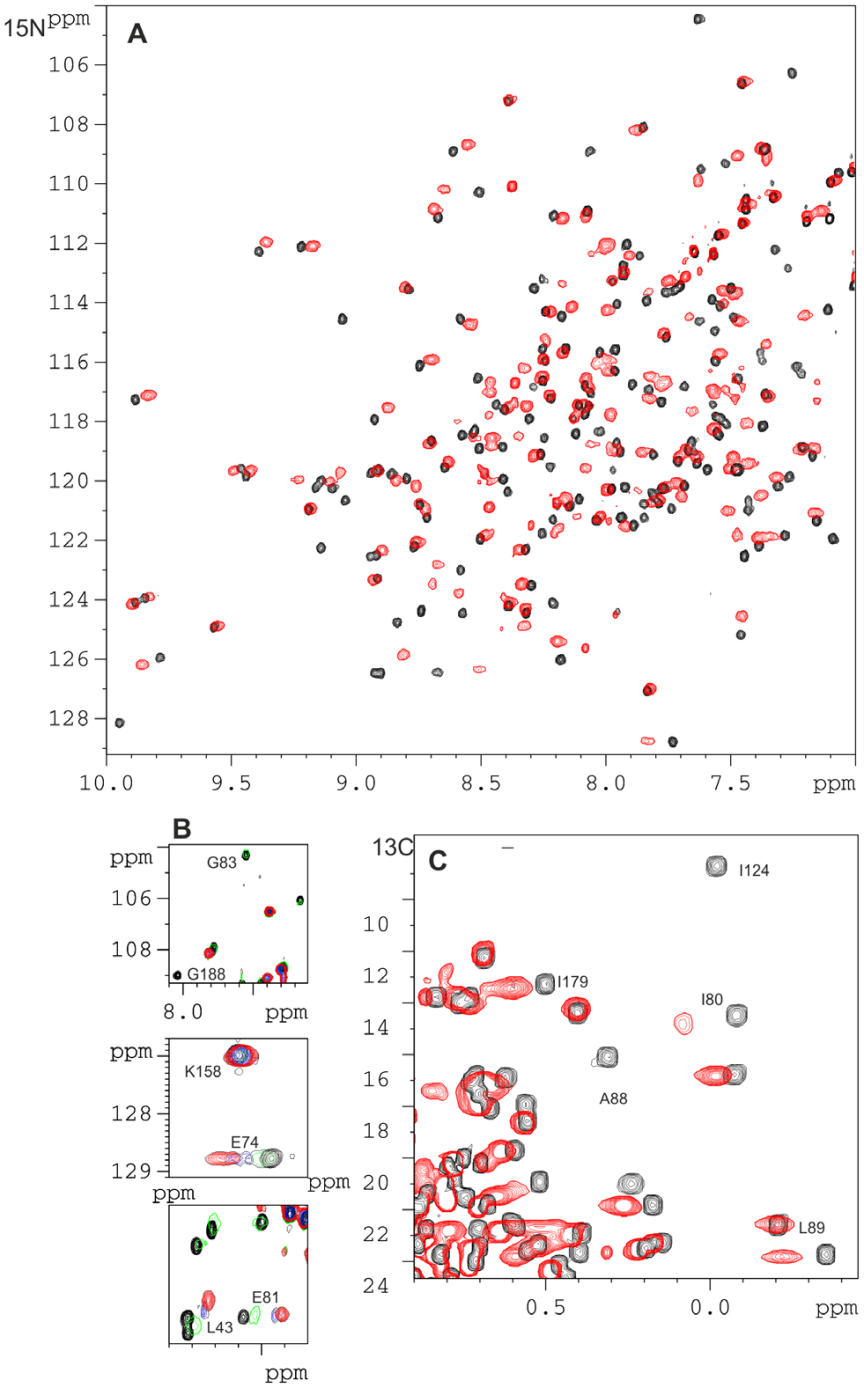
Interactions between NCS-1 and D2 Peptide monitored using NMR spectroscopy. (A) ^1^H^15^N HSQC spectra of ^15^N,^13^C NCS-1 (1 mM) in 50 mM Tris buffer, 50 mM NaCl, 5 mM CaCl_2_, pH 6.5, 300 K on Avance Bruker 800 MHz spectrometer in the absence (black) or in the presence of D2SR at a final concentration of 5 mM (red). (B) Sections of ^1^H^15^N HSQC spectra of ^15^N,^13^C NCS-1 showing the progression of peaks throughout the peptide titration (from black to red). Shown are the various characteristics of resonance perturbation ranging from complete line-broadening to gradual change in chemical shifts or a mixture of shifts and line-broadening. (C) ^1^H^13^C HSQC spectra of ^15^N,^13^C NCS-1 (1 mM) (black) in the presence of D2SR (final concentration of 5 mM) (red) indicating some of the resolved methyl groups with most significant shift changes.

NMR ^1^H-^15^N HSQC and ^1^H-^13^C HSQC spectra of ^15^N,^13^C-labelled NCS-1 were obtained. As described previously [43] the Ca^2+^ -bound form of NCS-1 showed sharper peaks compared to the apo-NCS-1. To identify the D2-binding surface of NCS-1, NMR ^1^H-^15^N and 1H-13C HSQC spectra of ^15^N, ^13^C-labelled NCS-1 in the presence of increasing concentrations of D2SR were obtained at two different temperatures (27^°^C and 35^°^C). At both temperatures chemical shift changes and/or broadening of specific NCS-1 resonances were observed (Figure 3). These perturbations were selective, suggesting that the NCS-1 remains a monomer when bound to the peptide.

**Figure 4:**
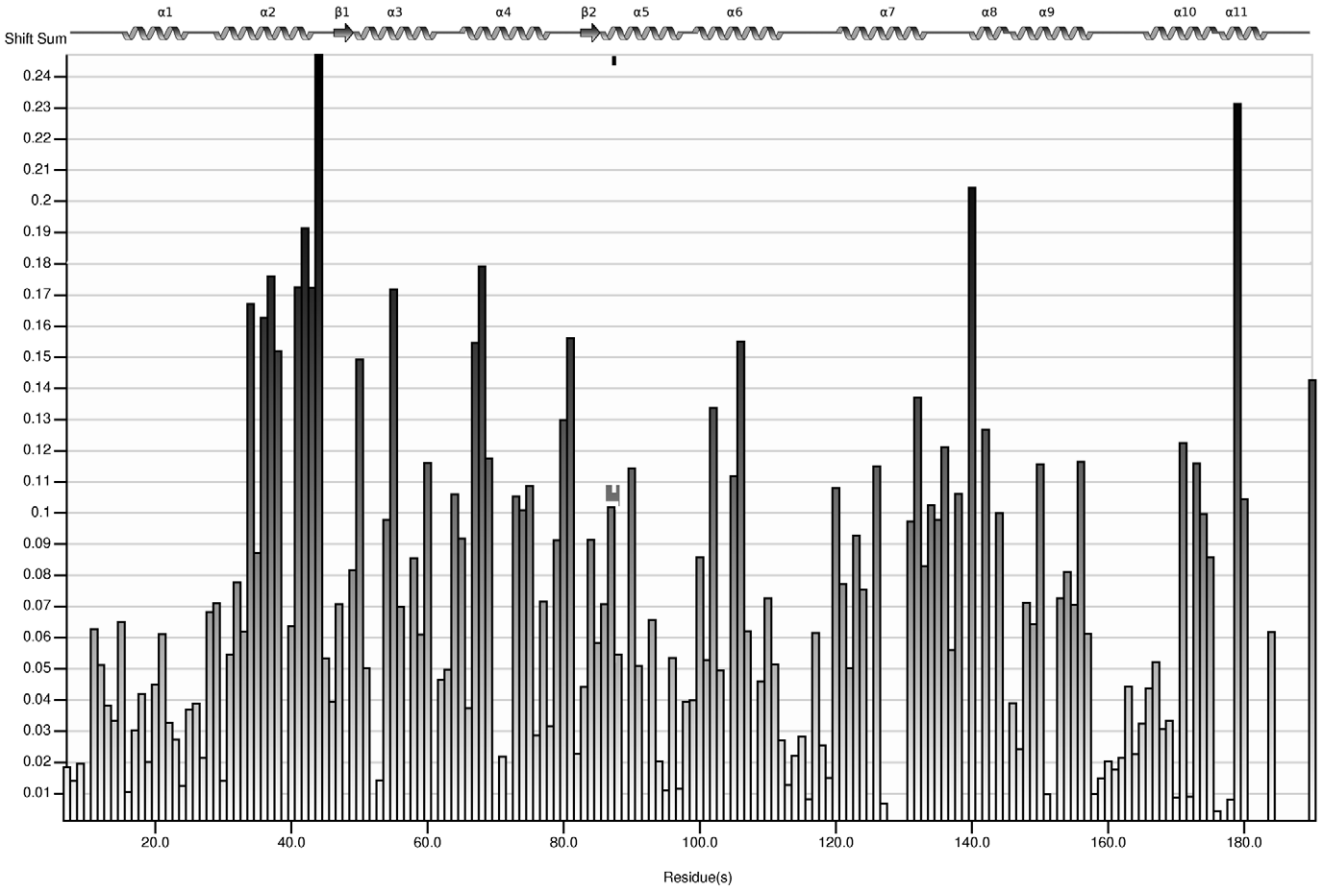
Histogram of chemical shift changes observed on D2 peptide binding to NCS-1. The shifts of NCS-1 in the presence and absence of D2 peptide (see Experimental Procedures) are compared and the shift differences expressed as Δδ = √ {(ΔH/0.03)^2^ +(ΔN/0.03)^2^}.The gaps are from either proline residues or due to the fact that the resonances for these residues very severely broadened and undetectable at 35^°^C; most significantly broadened residues are those from the C-terminus region (from residues 181) and in the unstructured region between residues 128–131.

**Figure 5:**
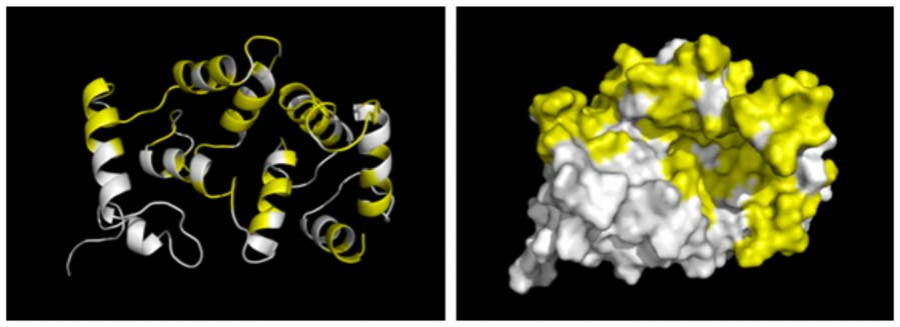
NMR-derived D2 binding site on NCS-1. (Left) Cartoon diagram of NCS-1 with residues that show significant changes in chemical shifts and/or line-widths coloured in yellow. (Right) Molecular surface of NCS-1 in the same orientation as the left-hand figure showing that many of the residues affected by the presence of D2SR are in or surround the hydrophobic crevice of NCS-1. The structures were created using the program Pymol (The PyMOL Molecular Graphics System, Version 1.3, Schrödinger, LLC).

The assignments of the resonances from the bound form were obtained by tracking the chemical shift changes over the titration experiment (Figure 4); many of the resonances showed an intermediate to fast exchange regime. When resonance perturbations are mapped onto the structure of NCS-1 (using Protein Data Bank structure 1G8I), the most affected ones are from residues located either in the hydrophobic ligand-binding crevice or in the regions linking the helical regions of the EF hands (Figure 5). The least affected regions of the protein are helices1, 5, 8 and part of 2; these regions form the periphery of the hydrophobic crevice and hence are unlikely to be involved with ligand binding. As shown in Figure 5, resonances of residues from both the N-and C-terminus hydrophobic binding sites are affected (coloured yellow). With the 2∶1 binding stoichiometry determined by fluorescence spectrophotometry, it is highly likely that both regions bind the D2 peptides. This would be consistent with the observed binding of two helices from Pik1 to Frq1 [31]. In the present studies, many of the broadened resonances remained undetectable at 35^°^C and even when a three-fold excess of peptide (measured using a 2∶1 stoichiometry of peptide: NCS-1) was present. The severe line-broadening of both the NCS-1 and peptide resonances precluded the determination of the structure of NCS-1-D2SR spectrum using conventional methods.

It is clear from the structure of the complex between Frq1 and Pik1 peptide that the N-terminal ligand binding site is more hydrophobic than the C-terminus site and this could affect the relative affinities of the site for the same ligand. Hence, the most appropriate model for NCS-1 interactions with the D2 peptide is a complicated two- site model where the binding sites are not identical. Furthermore, the severe line-broadening observed for several of the resonances also suggests the possibility of an internal exchange of the peptide between the two sites.

The pertubations of resonances from residues in the flexible linker region such as residues 138–145 would be consistent with conformational changes on peptide binding similar for those seen between unliganded Frq1 and the protein in the FRQ1/Pik1 complex [31], [32], [44]. This would suggest that NCS-1 undergoes a similar conformation change on binding D2 peptide as seen for Frq1 and that the ligand- bound NCS-1 has a more open hydrophobic groove than seen in the crystal structure of unliganded NCS-1 [30].

Confirmation that hydrophobic residues from both the N and C-terminal ligand binding sites are involved with peptide interaction comes from the shift changes observed for the methyl resonances in the ^1^H-^13^C HSQC spectrum; for example, residues such Ile 80, Ala 88, Leu89, Val 124, and 179 are significantly affected by the presence of D2 peptide (Figure 3C). On the other hand the methyl groups of L16, Met155, 156 and Ala154 are not significantly shifted by the presence of the peptide. These results show that the shift changes caused by the presence of D2 peptide are selective.

**Figure 6:**
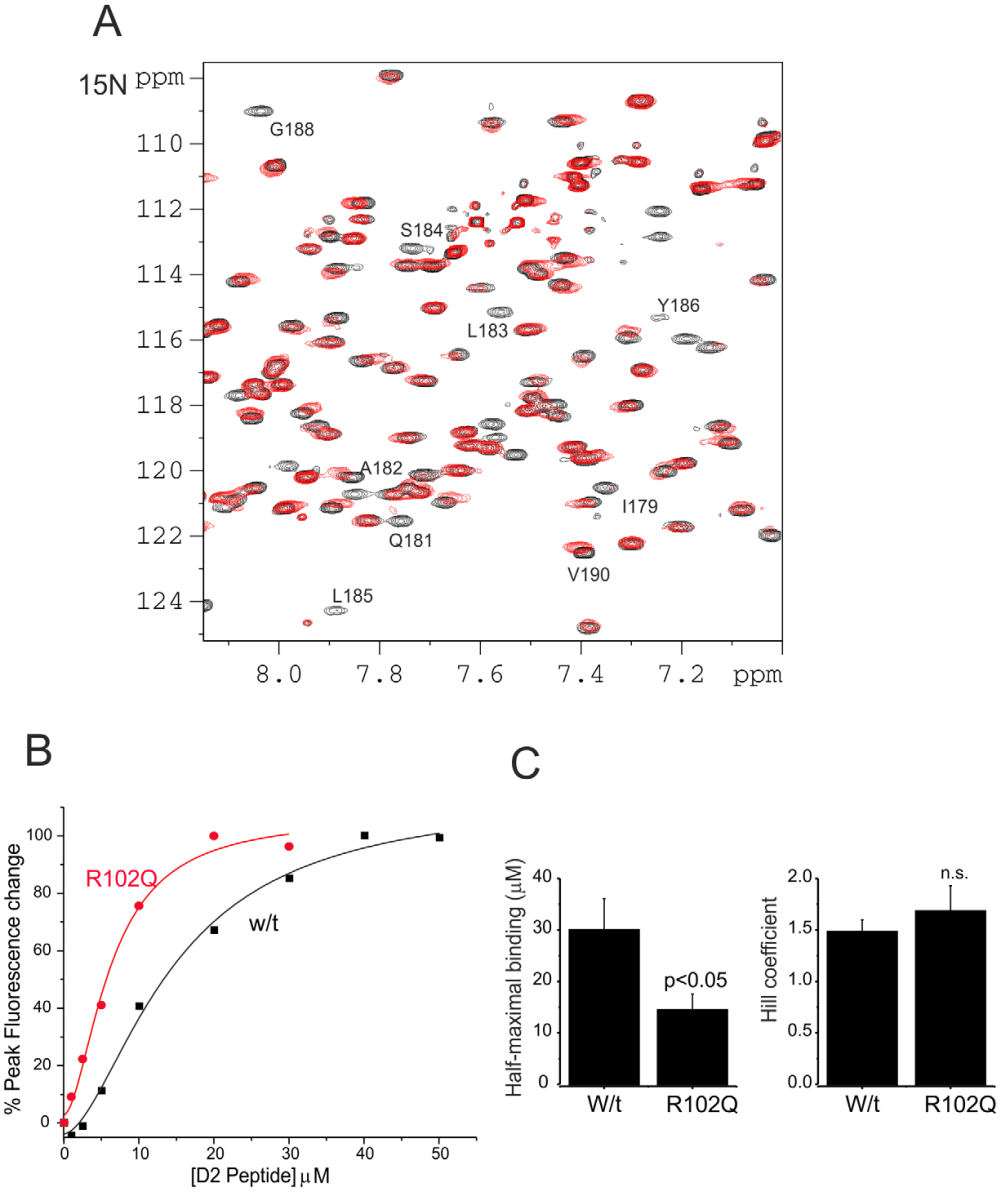
Significant line-broadening in the C-terminal region of NCS-1 and effect of the R102Q mutation on peptide binding. (A) Section ^1^H^15^N HSQC spectra of ^15^N,^13^C NCS-1 (1 mM) in the absence (black) or in the presence of D2SR (final concentration of 0.5 mM) (red) in 50 mM Tris buffer, 50 mM NaCl, 5 mM CaCl_2_, pH 6.5, 300 K on Avance Bruker 800 MHz spectrometer. At these initial stages of peptide titration, specific line-broadening of many residues from the C-terminal region was observed. (B) Extent of the changes in tryptophan fluorescence following sequential additions of D2SR peptide to the final concentrations indicated in the presence of 1 µM Ca^2+^. The data were fitted using a logistic fit by non-linear curve fitting. (C) Curve fitting of data from separate tryptophan fluorescence experiments enables determination of the concentration of D2SR peptide required for half-maximal binding and the Hill coefficient for wild type and R102Q NCS-1. The data are shown as mean±SEM from 5 (wild type) or 4 (R102Q) experiments and were compared using an unpaired Student's t-test.

Interestingly, resonances of residues from the C-terminus residues 180–190 which include residues from the C-terminal helix (helix 11 in Figure 1, helix J in the crustal structure [30]) are severely broadened throughout the peptide titration to the extent that some are not detectable at all (Figure 6A) even in the presence of a large excess of peptide. The resonances from this region were the first ones to be affected in the course of the peptide titration, with the line-broadening occurring at sub-stoichiometric concentrations of D2SR. The data suggest that the C-terminal region becomes disordered upon peptide binding; this is an indication that the C-terminal helix J is displaced to allow exposure of the hydrophobic cleft in order for the peptide to bind. This makes sense since in the crystal structure of NCS-1 (PDB accession number 1G8I), helix 11 occupies the C-terminal hydrophobic binding groove, effectively blocking any ligand interaction. Disorder of the C-terminal region was also observed in the Frq1-Pik1 complex [31].

To test the prediction that movement of the C-terminal region is important for ligand interactions, we examined binding of D2SR peptide to the autism-related R102Q mutant form of NCS-1. We have previously shown that the mutation results in a loss of resonances from amino acids in the C-terminal helix 11 in the ^1^H-^15^N HSQC spectrum of the of ^15^N-labelled R102Q NCS-1 protein consistent with increased dynamics of this C-terminal region [18]. It could be predicted, therefore that the R102Q mutation should increase the accessibility of the hydrophobic groove in NCS-1 for D2 peptide binding. A direct comparison of D2SR binding to wild type and R102Q NCS-1 was carried by monitoring changes in tryptophan fluorescence in parallel. The fluorescence spectra for wild type and R102Q NCS-1 were essentially identical and similar fluorescent changes occurred upon Ca^2+^ addition with the proteins having similar affinities for Ca^2+^. Addition of D2SR to either protein increased the level of tryptophan fluorescence (Figure 6B). A peptide titration indicated that in each case the data could be fitted to a curve indicating a Hill coefficient of around 1.5 and that the R102Q mutation increased the affinity of the protein for D2SR by 2.07-fold (Figure 6B,C). This finding is consistent with the prediction made above.

### Modelling of D2/D3 peptide and D3 receptor binding to NCS-1

**Figure 7:**
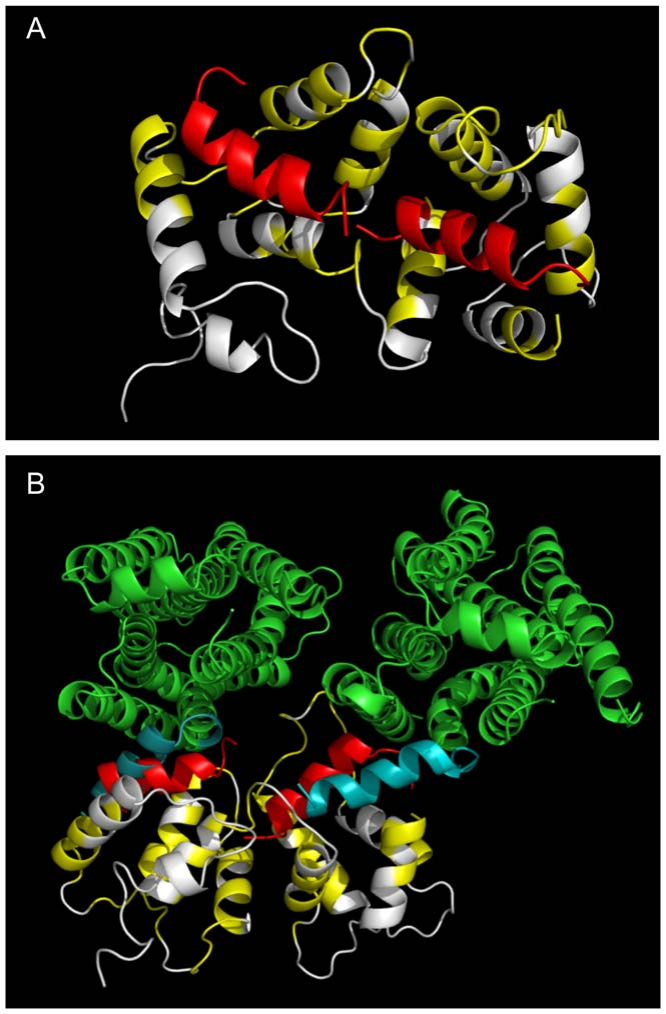
A proposed model of the complex between NCS-1 and the D2/D3 receptor. (A) Model structure of NCS-1 with C-terminal peptide TFNIEFRKAFLKILSC. NCS-1 residues with backbone and side-chain chemical shifts that undergo more than 0.07ppm chemical shift changes or which are completely broadened are coloured yellow. Unaffected NCS-1 residues are coloured white. The C-terminus D2/D3 peptide (coloured red) were simultaneously docked onto NCS-1 using HADDOCK 2.1 [41]. (B) Structure of NCS-1 with the D3 receptor. This structure is superimposed on the NCS-1-peptide complex described in (a). The receptor is coloured green with its C-terminus coloured teal. The model shows that it is possible to dock two receptors into NCS-1, with the C-terminus peptide placed in similar locations as when using the isolated peptides.

We tested the hypothesis that two D2 peptides would bind to NCS-1 once it had undergone conformational changes similar to those seen in the Frq1/PIK1 complex. This was done by docking residues 385–400 from the crystal structure of the dopamine D3 receptor (PDB 2PBL) onto NCS-1. From the modelled structure (Figure 7A) it is clearly possible that two D3 peptides could be accommodated in a conformation where the chemical shift perturbations are satisfied. In this structure, the C-terminal cysteines are located towards the centre of NCS-1, with the first phenylalanine residue not in contact with the protein. This model concurs with our preliminary finding that the shorter peptide TFNIEFRK is not able to bind NCS-1. The orientation of the peptide in the C-terminal hydrophobic site corresponds with that observed for the Pik1 (residues 156–170) interaction with Frq1 [31], and the way the C-terminus of NCS-1 and Frq-1 occupy this binding site in the structure of unliganded proteins (PDB accession 1G8I and 1FPW).

NCS-1 has also been shown to bind to the dopamine D3 receptor and to regulate its internalisation [25]; the D3 sequence differs by only one amino acid from D2. Ultimately, it would be important to understand how NCS-1 interacts with the intact receptor. We, therefore, constructed a model of the NCS-1-D3 receptor complex using the AIR derived from the D2SR peptide-NCS-1 chemical mapping data, and taking into consideration that many of the hydrophobic residues at the C-terminus of D3 receptor are involved in intramolecular interactions with helix I of the receptor [45]. HADDOCK docking produced a plausible model shown in Figure 7B. It is possible to sterically accommodate two receptors docked onto NCS-1, with the C-terminal region located in similar regions of NCS-1 as the isolated receptor peptides. Subtle changes at the junction of helices VIII and VII are required to achieve this complex structure. In addition, in the N-terminal hydrophobic groove of NCS-1, the bound receptor is orientated such that its C-terminal helix is in the opposite direction to that obtained when the peptide alone is used in the docking, whereas in the C-terminal groove, the peptide and receptor bind in similar orientations.

## Discussion

The positions of hydrophobic residues that are solvent exposed and form contacts with target peptides from Frq1, recoverin, and KChIP1 were mapped onto the NCS-1 sequence revealing the similarities in residues involved in the three characterised interactions and that these correspond to conserved hydrophobic amino acids in NCS-1 (Figure 1). The determined structures of the complexes reveal similarities and differences in the binding interactions. The N-terminus of rhodopsin kinase interacts through a single helix in an N-terminal hydrophobic pocket [33] and the C-terminal part of the hydrophobic pocket is occluded by the recoverin C-terminal J helix (referred to as helix 11 here, as shown in Figure 1). Recent evidence suggests that residues of the recoverin J-helix are directly involved in the interaction with rhodopsin kinase peptide [46]. In contrast, in the Pik1/Frq1 complex two helices of Pik1 are bound in each of the N- and C-terminal pockets in Frq1 [31]. The interaction of the Kv4.3 N-terminal domain with the hydrophobic cleft of KChIP1 [34], [35] involves similar N-terminal residues as seen in recoverin/rhodopsin kinase and Frq1/Pik1 complexes and also some residues part way into the region of the C-terminal pocket in Frq1 bound by the second helix of Pik1. The structure of Ca^2+^-bound NCS-1 shows the presence of a large hydrophobic groove stretching across residues equivalent to both N- and C-terminal pockets in Frq1 and in the crystal structure this groove is occupied by polyethylene glycols [30]. This structure could reflect the structure of NCS-1 as it would be after peptide ligand binding but it is also likely that peptide ligand binding could result in additional structural changes in NCS-1 that were not observed in the crystal structure.

We examined the possibility that the interaction of the dopamine D2 C-terminus with NCS-1 was structurally similar to that in the Frq1/Pik1 complex. Hydrophobic residues in NCS-1 that could be involved in target protein interactions within an exposed hydrophobic groove include the two tryptophan residues; the indole NH NMR resonances were severely broadened when the D2 peptide was present. In unliganded NCS-1, the C-terminal region including the helix J (helix 11 in Figure 1) forms one side of the large hydrophobic groove. When D2 peptide binds, the conformation of this region was severely perturbed, consistent with displacement of the J helix from the hydrophobic pocket as seen for the KChIP1/Kv4.3 complex. In the Frq1/Pik1 complex helix J was found to be unstructured [31]. In the KChIP1/Kv4.3 structure the C-terminal helix J was found to have undergone a large conformational change compared to KChIP1 to create the binding site [35]. The structure of KChIP1 in the complex was comparable to NCS-1 in the crystal structure where helix J was displaced by 45^0^ compared to the corresponding helix in the un-liganded neurocalcin structure [47]. The loss of NMR amide resonances from residues in the J helix (helix 11) suggests that on D2 peptide binding, this region becomes highly dynamic; interestingly these resonances were affected when substoichiometric amounts of the D2 peptide was present, providing evidence that movement of helix J is an early event in D2 peptide binding. In addition, even in recoverin where the significant displacement of helix J does not occur on ligand binding this helix was found to be dynamic suggesting transient flickering between states [48]. We, therefore, suggest a model in which D2 peptide binding to NCS-1 leads to conformational changes including movement of the J helix to maximally expose the hydrophobic groove. A similar loss in resonances from residues in the J helix was seen in NCS-1 bearing an autism-related mutation (R102Q) that was predicted to disrupt hydrogen binding between helix F and helix J [18]. To test the idea that movement of the C-terminal helix may be important to allow D2 peptide binding we compared the binding of the D2RS peptide to wild-type NCS-1 and the R102Q NCS-1 in which the C-terminus was already highly dynamic. This demonstrated that the affinity of binding was increased in the mutant supporting the model for the behaviour of the C-terminal helix.

Biophysical analysis of NCS-1 was consistent with the protein being monomeric in the presence or absence of Ca ^2+^. The data from spectrophotometry upon D2 peptide binding giving a Hill coefficient for the binding interaction of greater than 1 would be consistent with a 2∶1 stoichiometry of D2 peptide binding to NCS-1 suggesting that two molecules of the D2 peptide could bind within the large hydrophobic groove of NCS-1. The D2 peptide is predicted to be alpha-helical and the C-terminus of the dopamine D3 receptor which can also bind to NCS-1 [25] and differs by one only residue from the D2 peptide forms an amphipathic alpha-helix in the crystal structure of the D3 receptor [45]. We suggest that two such alpha-helices could bind within the NCS-1 hydrophobic groove similarly to the two helices from Pik1 that bind to Frq1 [31]. The NMR resonance shift data are consistent with conformational changes on D2 peptide binding being similar to those in the Frq1/Pik1 complex that would allow this. A HADDOCK model of the NCS-1-peptide complex using ambiguous interaction restraints derived from the NMR data could be successfully obtained. These models show that the N-terminal phenylalanine residue in the peptide is not involved in NCS-1 binding and this concurs with the binding assays which showed that the shorter 8-residue peptide (TFNIEFRK) was not able to bind NCS-1.

Interestingly, Dopamine D2 receptors function in signalling as homodimers [49], [50], [51]. This suggests a possible model in which a single NCS-1 molecule is its Ca^2+^ bound state is functionally associated with both partners of the dimer. We used similar experimental restraints to drive the docking of two molecules of the D3 receptor onto NCS-1 which produced a model in which the C-terminus of the each receptor molecule is located in similar regions of NCS-1. To accommodate two receptors with minimal steric clashes, conformational changes around the hinge region between helices VIII and VII of the receptor are necessary, together with a change of the orientation of the C-terminal peptide in the N-terminal hydrophobic groove of NCS-1.

Overall the conclusion from this study are consistent with NCS-1 fitting a general model for target protein interactions by the NCS protein family, This would involve binding of one or more alpha-helices of the target within an exposed hydrophobic domain. Specificity of interaction with NCS proteins would be determined by the unique sequences and structurally different dynamics of the C-terminal helix.
